# The role of fines in espresso extraction dynamics

**DOI:** 10.1038/s41598-024-55831-x

**Published:** 2024-03-07

**Authors:** Samo Smrke, André Eiermann, Chahan Yeretzian

**Affiliations:** 1 Institute of Chemistry and Biological Chemistry, Coffee Excellence Center, Zurich University of Applied Sciences, Einsiedlerstrasse 31, 8820 Wädenswil, Switzerland; 2 Independent researcher, Sierre, Switzerland

**Keywords:** Coffee, Grinding, Extraction, Espresso, Applied physics, Analytical chemistry, Mass spectrometry

## Abstract

The impact of particle size distribution of coffee grounds on espresso extraction was explored. Finely ground coffee for espresso has a characteristically bimodal particle size distribution. For a given median grind size, different grinding technologies can yield a different share of fines (particles < 100 µm). We performed espresso extractions for a range of median particle sizes and systematically varying the share of fines by adding sieved fines to the coffee grounds. Dynamic beverage weights, extraction percentage, extraction time and dynamic headspace PTR-MS (proton-transfer mass spectrometer) analysis and sensory evaluation of the resulting brews were measured. We show that the share of fines plays a key role in the espresso extraction flow rate. An increase of share of fines decreases coffee bed permeability, leads to reduced flow rates and longer extraction times. A statistical model using partial least squares regression of the particle size distributions of coffee grounds confirms that fines decrease the coffee bed permeability. The PTR-MS analysis shows a non-linear increase of aroma compounds in the cup with increasing extraction yield. Our hypothesis is that both extraction efficiency and post-extraction evaporative losses of aroma compounds influence the final aroma compound concentrations in the cup.

## Introduction

Espresso beverage extraction has been widely studied and it is well known that a successful extraction of an espresso depends on a range of extraction variables^1–6^. One of the most varied settings when extracting espresso is the grind size of the roasted and ground (R&G) coffee. The optimal grind size depends on the type of coffee used, blend, age of coffee, and consumer preferences^2^. Grind size might be adjusted numerous times during the day in a café as the environmental conditions (humidity, temperature) change.

The effect of R&G grind size on coffee extraction has been described extensively in the literature^7–17^. Particle size is often described by a single number, which might be as simple as an arbitrary grind size setting on a grinder, or as a volume weighted average (or median) particle size calculated from the particle size distribution. Particle size of R&G coffee impacts extraction dynamics of espresso, as finer grind size creates a larger pressure drop^18,19^ and consequently due to 9 bar standard espresso extraction pressure^2^ leads to longer espresso extraction time. Solubles will extract from finer ground coffee particles faster, however the transport from the bed will be slower due to lower flow rates, which in consequence does not necessarily make fine grind the most efficient extraction^20^. Furthermore, when grind size is too fine this can lead to uneven extraction and lowering of the extraction yields^14^.

It is known that permeability of packed beds for different materials will depend on parameters such as particle size, shape, size distribution, packing arrangement and roughness^21^. Among all those parameters the particle size of the ground coffee has attracted most attention in studies on coffee extraction. However, R&G coffee is well known to have a bimodal particle size distribution. Model studies have considered the bimodal distribution^13,18^, but the variable nature of the distribution has not been studied specifically. Permeabilities of beds comparable to coffee are complex to model, hence behavior of packed beds composed of irregular particles could only be predicted by empirical models^21^. In our previous study about capsule coffee extraction, we found that projecting a full particle size distribution into a single particle size number (e.g. the median) does not allow to characterize sufficiently the extraction dynamics of coffee capsules^22^. We identified a parameter that we termed the “share of fines” as a volume share of particles smaller than 100 µm^22^. It was found that the share of fines has a stronger impact and predictive power on capsule coffee extraction time than the overall average particle size.

This study explores the extraction dynamics of espresso extraction as impacted by different particle size distributions. The bimodal particle size distribution and the ratio of contributions of two peaks depends on the average particle size^23^ but is also indicative of the grinding technology^22^. Here, we used only a single grinder type and added various amounts of fines after grinding, which have been sieved beforehand to study the impact of fines on extraction of espresso coffee.

## Materials and methods

### Coffee, grinding and preparation of fines fraction

A single origin *Coffea arabica* L. from Costa Rica, processed as a pulped natural post-harvest process was used. The roasted coffee was obtained from a local roaster (G. Henauers Sohn AG, Höri, Switzerland), and was roasted to a roast color of 143 Colorette Pt, measured by a Colorette 4 (Probat AG, Emmerich, Germany). Samples were ground using a Bentwood Vertical 63 grinder (Bentwood GmbH, Cham, Switzerland) at various grind size setting. Vertical 63 grinder has a scale that is calibrated to the grinder burr spacing in µm; these burr spacing values are reported here as grinder settings. The sample of fines was prepared in advance to coffee extraction. A sieve of 120 µm size (Retsch GmbH, Haan, Germany) was used to obtain the fines fraction from finely ground coffee.

### Preparation of R&G samples, and espresso extraction

R&G samples were prepared by grinding 20 g of coffee at grinder settings of 160, 170, 180, 190, 210, and 250. For each of samples at 190, 210 and 250, a fines fraction was added, by adding 1, 2, or 4 g of the fines fraction to 19, 18, or 16 g of coffee, respectively. This was done using the following protocol: the whole beans are filled into an empty Vertical 63 grinder and the whole dose of coffee is ground into an espresso preparation tool Blind Shaker (Weber Workshops, Incline Village, Nevada, USA). Then a pre-weighed amount of fines fraction is added to the coffee and shaken in the tool to homogenize the sample. Finally, ground coffee was transferred to a 20 g espresso portafilter basket (VST inc., Tulsa, US), and tamped using 20 kgF.

For all coffee extractions 20 g of R&G coffee was extracted on a Victoria Arduino Black Eagle coffee machine (Simonelli Group SpA., Belforte del Chienti, Italy) at 9 bar pressure and manually controlled to yield 40 g of beverage. The weight of the beverage during the extraction was monitored and recorded using a load cell. The extraction time as displayed by the espresso machine was used. The procedure described in this section (preparation of R&G samples and extraction) was performed in 3 replicates.

### R&G coffee and beverage analysis

Particle size analysis was performed using an imaging particle size and shape analyzer with a dual camera system Camsizer X2 (Retsch Technology GmbH, Haan, Germany). At least 10 g of coffee was used for each particle size analysis, and three measurements were performed per sample. Median particle size (X50) was determined as the volume weighted median particle size, based on the projected particle area on the images. The share of fines (Q_100µm_) was defined as the volume share of particles that are smaller than 100 µm in size.

The concentration of coffee in the filtered brew (0.45 µm filter) was analyzed using a VST LAB Coffee III refractometer (VST inc., Tulsa, US), which is calibrated to display total dissolved solids (TDS) in coffee brews. The extraction percentage (extraction yield) was calculated based on the weight of the coffee brew and refractometric TDS measurement.

The sensory test of coffees was performed by a Q-grader (certified Q Arabica Grader by the Coffee Quality Institute). The sensory parameters were determined as hedonic scores for flavor, balance and tactile on a scale from 0 to 5. Sensory was meant to be used only as a subjective guidance for optimization of coffee extraction, as according to industry practice, and was not performed as a systematic double-blind sensory analysis test.

### PTR-MS analysis

**Figure 1:**
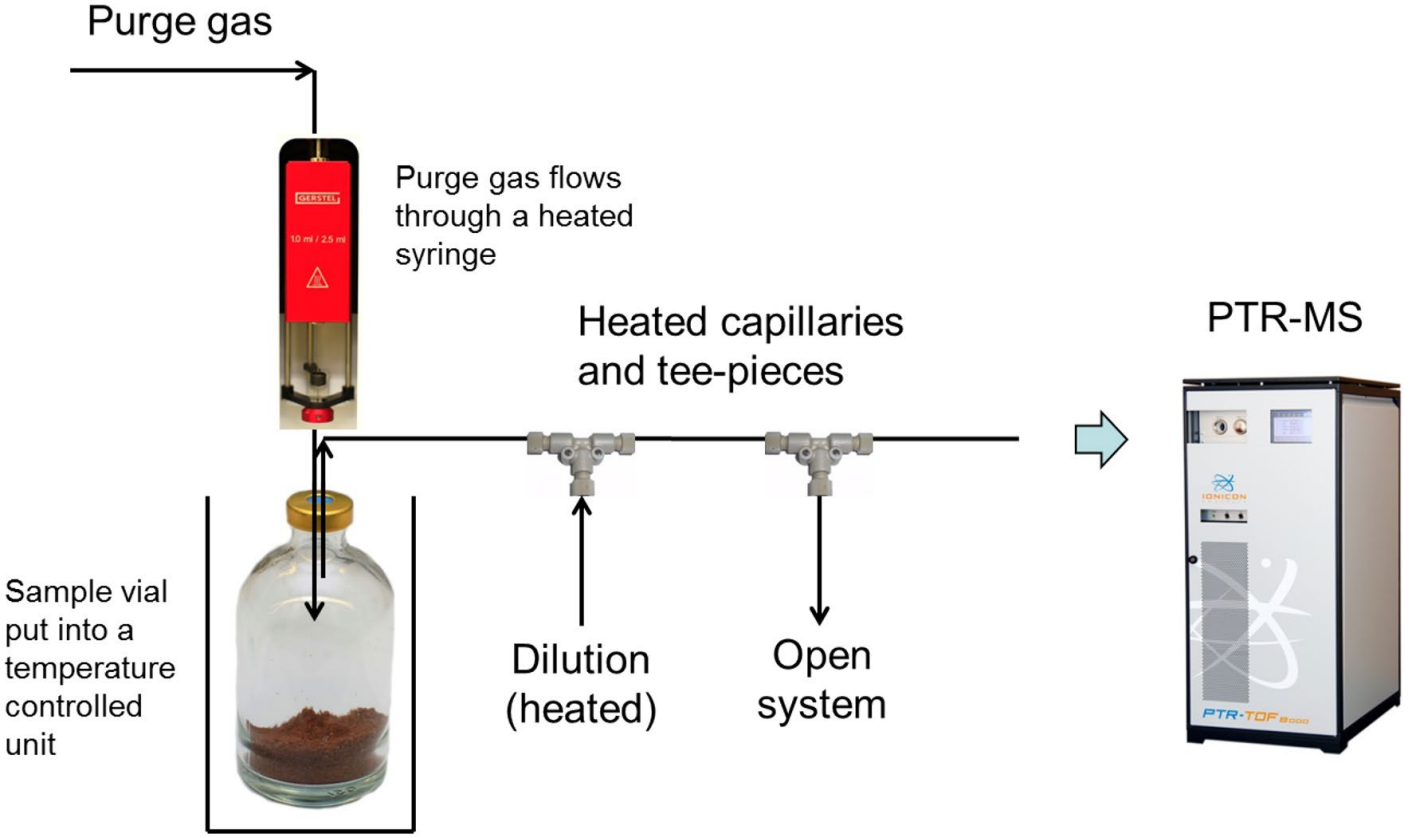
Schematic of the dynamic headspace proton-transfer mass spectrometry setup (DHS PTR-MS).

Proton-transfer mass spectrometer PTR-MS 6000x2 (Ionicon GmbH, Innsbruck, Austria) was used to determine the composition of the volatile organic compounds of the headspace above espresso samples. PTR-MS was coupled to an MPS autosampler using a PurgeXL sampling unit (GERSTEL GmbH & Co.KG, Mülheim an der Ruhr, Germany) to perform a dynamic headspace analysis. The schematic of the sampling setup is presented in Fig. 1. The headspace from a 20 mL vial, first preheated to 50 °C in an agitator unit, is sampled by nitrogen at 10 nmL/min from the Purge XL unit at 50 °C. The flow is then diluted with a nitrogen flow at 100 nmL/min and sampled using the PTR-MS. The excess of the flow is discarded though an open capillary exhaust at the back of the unit to keep the 1:10 dilution stable.

PTR-MS conditions used for the analysis were the following.

The raw data was processed using the IDA Ionicon Data Analyzer (Ionicon GmbH, Innsbruck, Austria), to obtain normalized counts per second (ncps) signals averaged in 5 s intervals, that is linearly related to VOC concentration. The signal in the interval 50–75 s after starting PTR-MS measurement (dynamic headspace conditions) was averaged and used for the analysis.

### Data analysis and statistical analysis

All data analysis and statistical analysis was performed using R statistical computing (R Foundation for Statistical Computing, Vienna, Austria) using integrated packages. The grouping of the PTR-MS measurements was done according to analysis of linear models of PTR-MS signal intensity as a function of coffee extraction yield. Group A: k > 0 and p < 0.05; Group B: k < 0, p < 0.1 for yield below 19.5% and k > 0, p < 0.1 for yield above 19.5%; Group C: all p values, for all yields and separate for below and above 19.5%, p > 0.05; Group D: k < 0 and p < 0.1. Partial least squares regression (PLSR) model was generated using the “pls” R-package version 2.8-1.

## Results and discussion

In a previous study^22^ we examined the relation of R&G coffee particle size distribution with extraction using commercially available single-serve Nespresso of Nespresso compatible capsules. This study develops upon the previously published work on capsules but uses espresso extraction with a professional machine as the extraction platform. By using professional espresso machine, we achieve higher control and consistency for the coffee and the extraction variables, allowing to systematically explore the relationship between PSD and resulting extraction dynamics and brew properties.

**Figure 2:**
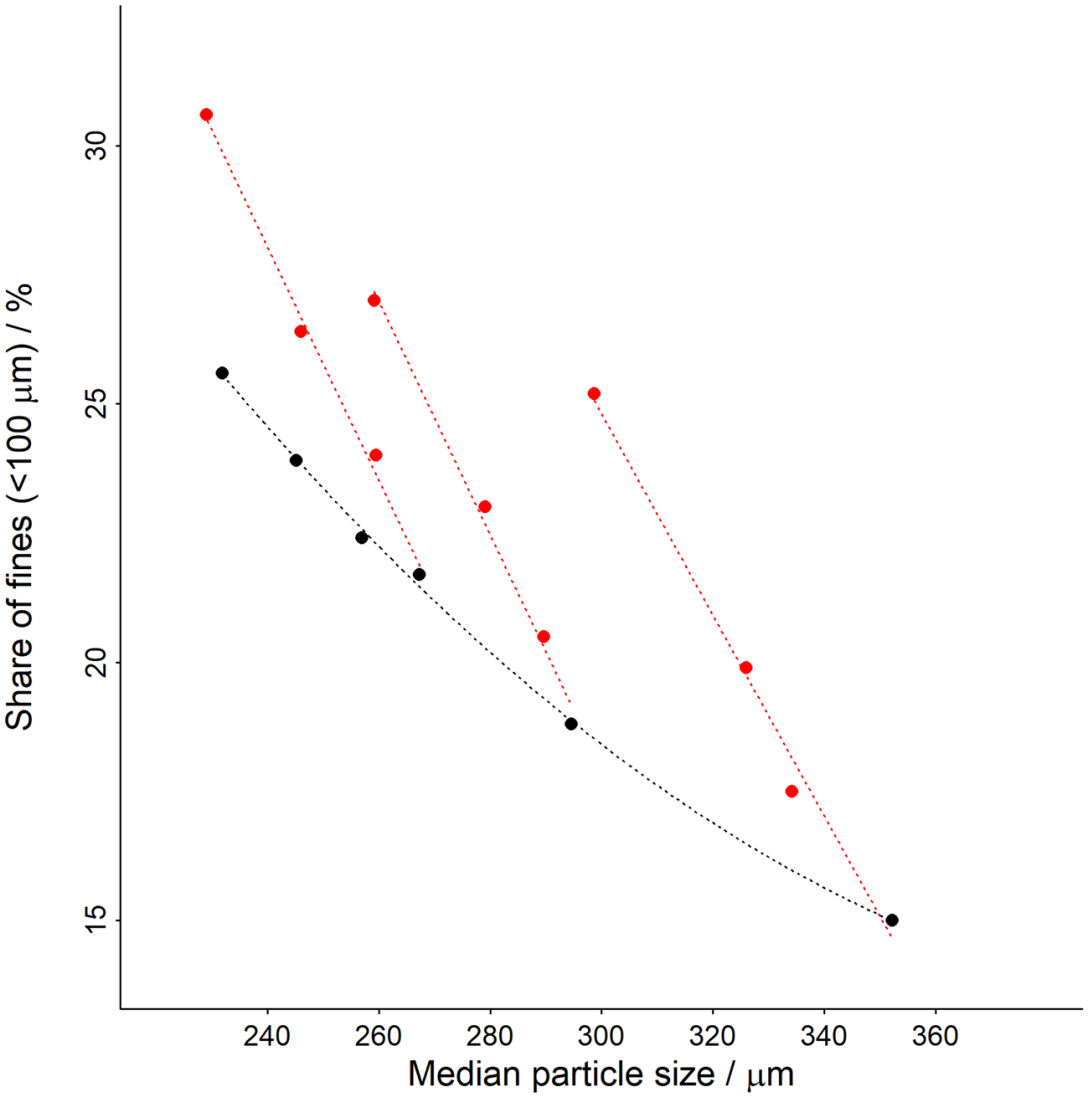
Relation of share of fines with the median particle size for the ground coffee samples. Black points are samples without added fines and red points are samples with added fines.

As described in the materials and methods section the coffee samples were manually spiked with fines before the extraction to generate a set of combinations of X50 and Q_100µm_ (Fig. 2). The resulting parameters extracted from the particle size distributions show a polynomic relation (second order polynomial fit displayed on Fig. 2 in black) of the fines fraction to the grinder burr spacing for samples without added fines. For samples with added fines, we observe as expected a linear relationship of the increase of Q_100µm_ with addition of fines and a small decrease in the X50 caused by overall smaller median particle size.

### Impact of fines on espresso extraction

**Figure 3:**
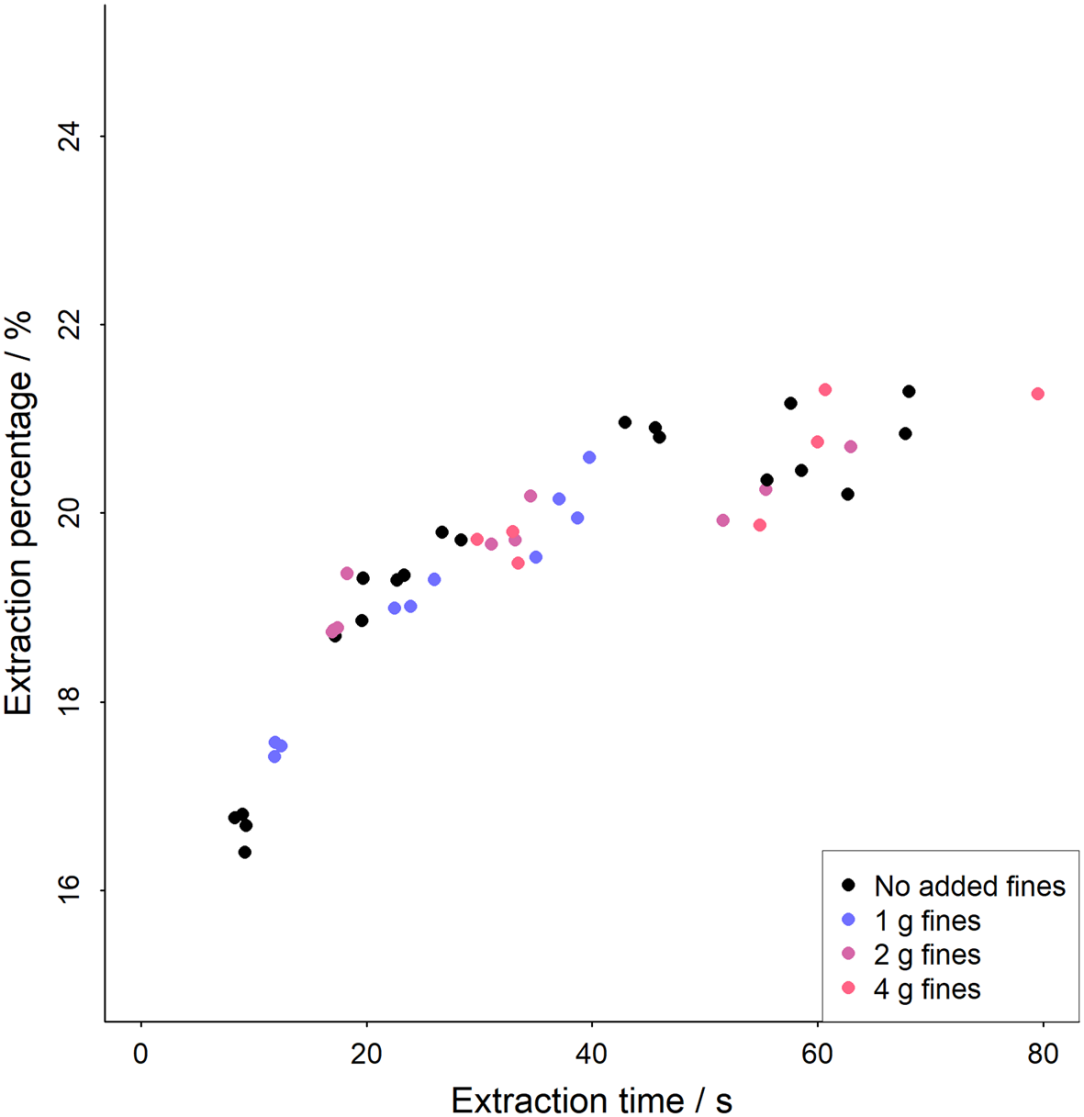
Extraction percentage (yield) of espresso extraction from coffee with varying particle size distributions. Different colors of the plot denote the amount of fines added to ground coffee before extraction.

During a typical espresso extraction around 20% of the solid coffee material is solubilized and extracted into the beverage (extraction yield). Figure 3 presents the extraction yield data for the coffees extracted in this study as a function of the extraction time. The result show that within a given margin error all measurement points are scattered around a curve. Addition of fines to the sample did not lead to outlying combinations of extraction yield/extraction time. We have not observed major changes in the flow rates of espresso extracted coarser with added fines, compared to those ground finer. A chart with flow rate profiles for all extraction can be found as Supplementary Fig. S1. Hence, we assume the amount fines does not fundamentally change the extraction mechanism and acts only by modifying the coffee bed permeability.

Maximum extraction yields reported in the literature differ widely and have not been yet studied systematically for a range of extraction method, water-to-coffee ratio, roast profile, and green coffee types. From the data in this study, we assume that for practical purposes within the given extraction recipe maximum extraction yield is reached for espressi with > 40 s extraction time.

The extractions conducted in this study confirm what has been discussed previously in the literature about efficient espresso extraction^18^. Fast espresso extractions are very efficient, as extractions of < 10 s and 15 s yielded 17–18% extraction, which is > 80% of maximum espresso extraction yield observed here. Such extraction has been gaining popularity and is colloquially known as “turbo” espresso by using extractions of 10–20 s instead of traditional 25–30 s.

### Statistical modelling of espresso extraction dynamics with R&G particle size distribution

**Figure 4:**
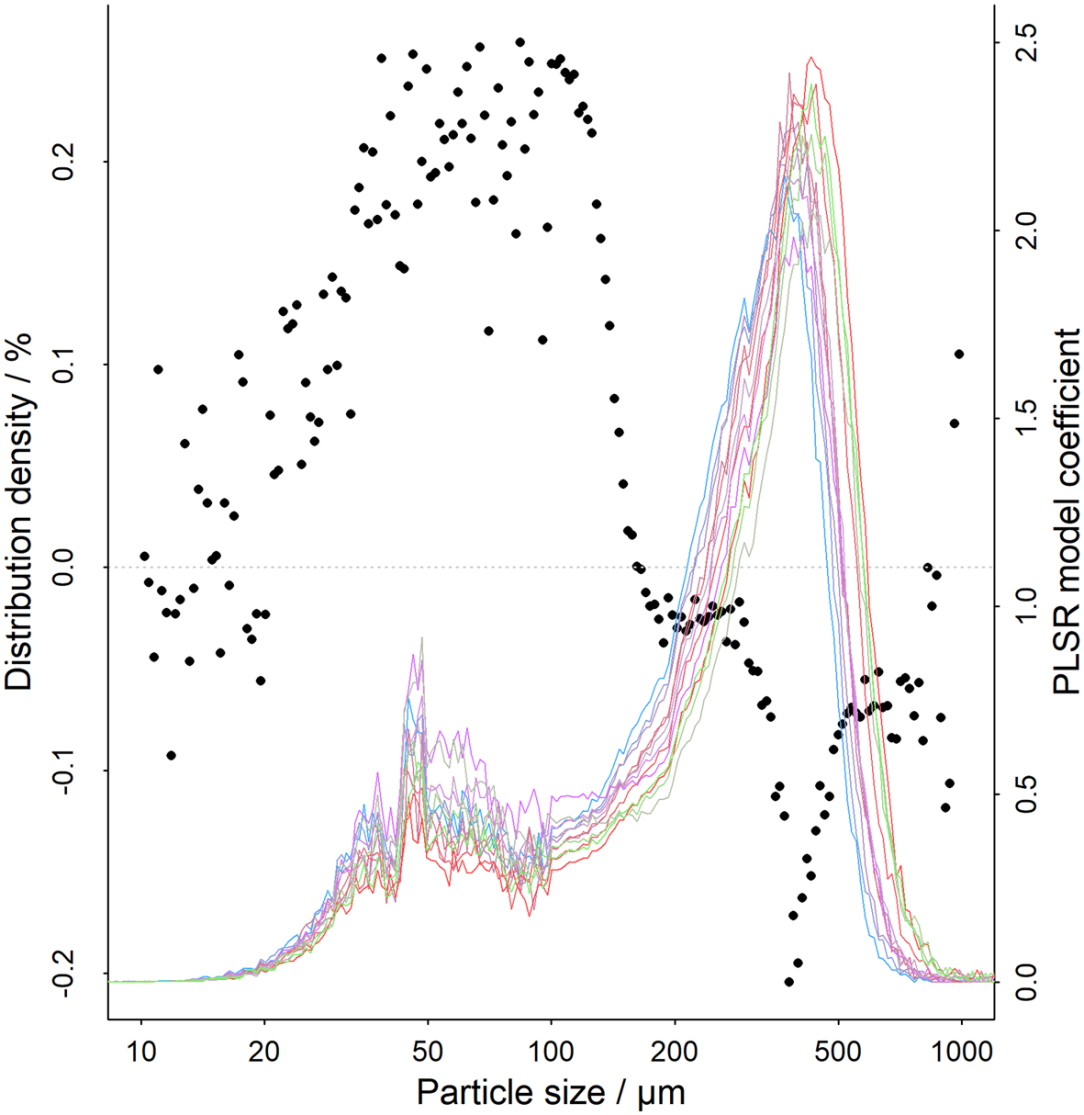
Partial least squares regression model coefficient (points) predicting espresso extraction time based on input variable of particle size distribution data (lines).

Measured extraction times of the espresso extracted with varying particle size distributions have been modelled using statistical methods. A Partial Least Squares Regression (PLSR) model was developed to predict extraction time using data from PSDs. The resulting model coefficients provide an insightful information regarding the impact of different parts of PSD on the extraction time (Fig. 4). PLSR coefficients are positive in the range of until 150 µm, have a zero value until 250 µm and a negative value for larger particle sizes. This result demonstrates that an increase of the share of fines increases extraction times. A higher share of the main peak, and in particular its shift toward values above 250 µm decreases extraction times. This is an expected result from the experience of studying espresso, however, it is of our knowledge the first time that such analysis has been conducted to confirm this phenomenon by using untargeted statistical analysis of whole PSDs.

**Figure 5:**
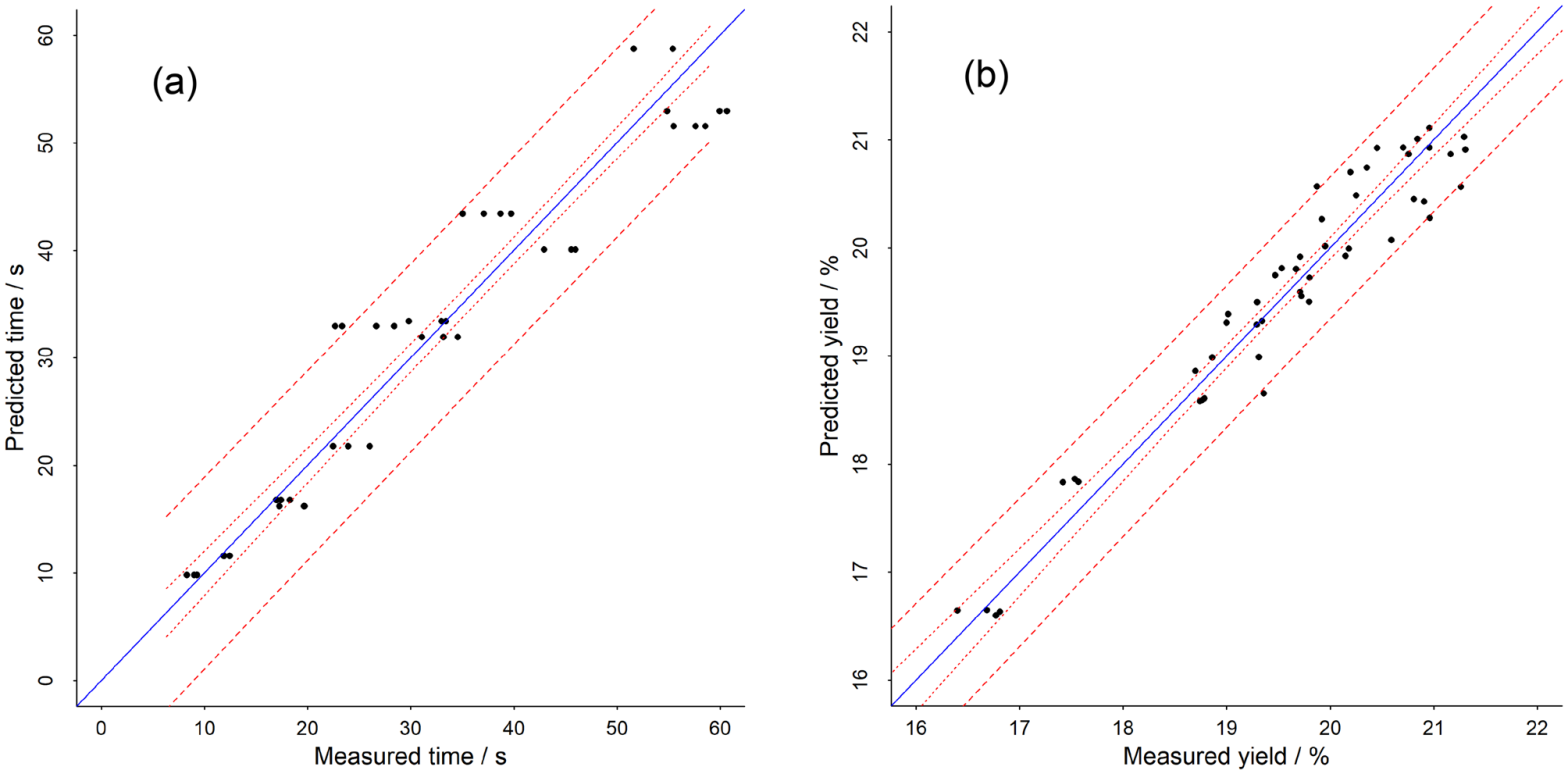
Multiple linear regression model performance for predicting espresso extraction time based on median particle size and share of fines (**a**); and predicting extraction yield based on median particle size, share of fines and extraction time (**b**).

A more precise model to predict espresso extraction time can be obtained by using multiple regression analysis with second order polynomial regression and using Q_100µm_ and X50 as input variables. Figure 5a shows the measured extraction times against the extraction times predicted by the model. Both variables, Q_100µm_ and X50 are significantly contributing to the model, with similar sized normalized model coefficients. This methodology can be further expanded to predict extraction percentage of an espresso extraction (Fig. 5b) by using Q_100µm_, X50 and extraction time as model variables. Reported studies have focused on fundamental modelling of espresso extraction dynamics^5,24^, and those give a fundamental view into the extraction mechanism. The models presented here have an applied target and are meant to model the parameters that can be impacted in a coffee shop type of espresso preparation situations.

### The impact of extraction variables on espresso VOC composition

Extraction yield is just one quantity of an espresso. Most of our perception of the flavour of the beverage stems from the composition of VOCs in the brew, the aroma^25^. We have analyzed headspace composition of espressi, by rapid direct injection mass spectrometry. The method enables high throughput of the samples (5 min/sample) and highly reproducible measurements for high-volatile VOCs. A disadvantage of direct injection PTR-MS is the identification of compounds. The identification was tentative based on molecular formula, known composition of coffee as measured by GC/MS, and previously published data^6,26^.

**Figure 6:**
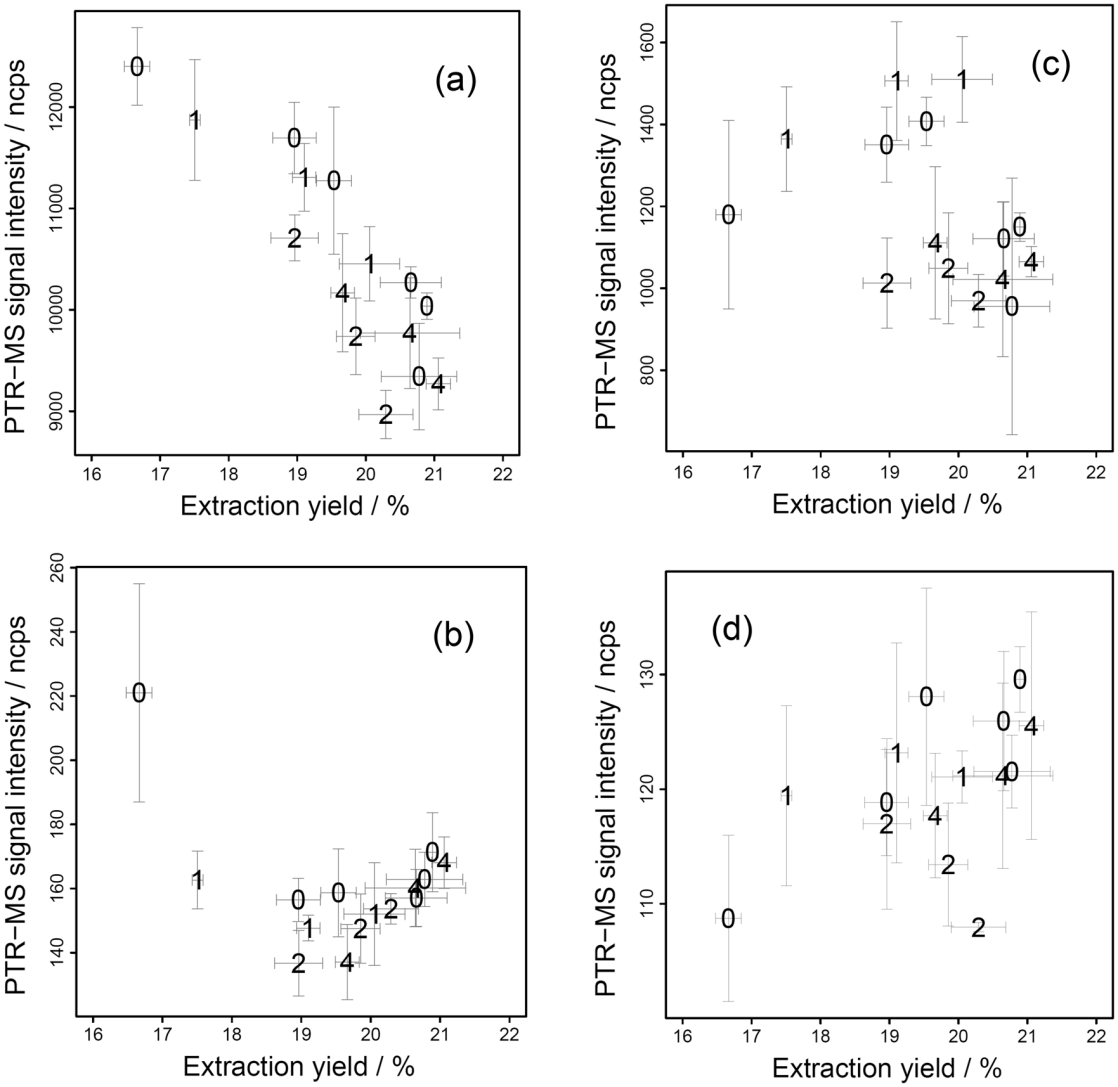
Measurement of the VOC signal intensity by PTR-MS from the headspace above espresso coffee sample, as a function of extraction yield impacted by extracting ground coffee with a varying particle size distributions. The numbers in the charts denote the amount of fines added to the coffee (in grams), the error bars are single standard deviation. (**a**) m/z = 75.042, tentatively indentified as methyl acetate, (**b**) m/z = 121.068, tentatively identified as ethyl-methylpyrazine, trimethylpyrazine, (**c**) m/z = 49.010, tentatively identified as methanethiol, (**d**) m/z = 127.034, tentatively identified as maltol, methyl furoate.

Selected representative PTR-MS signal intensities are presented as a function of the extraction yield (Fig. 6). We have found that VOCs group based on their properties and show characteristic behavior by their groups, similar as previously found in other studies^6,27^. For tentative identifications of m/z values refer to Sanchez-Lopez et al.

Group A (example Fig. 6a): compounds that concentration continuously decrease with increasing extraction yield. Selected PTR-MS peaks showing this behavior are (experimental m/z): 31.020, 33.036, 45.034, 47.013, 59.050, 61.028, 68.046, 69.032, 73.063, 75.044, 82.058, 87.037, 87.074, 89.055, 101.054, 113.052, 115.066, 127.080.

Group B (example Fig. 6b): compounds that show an initial decrease in VOC quantities for fastest extracting samples (with lowest yield), a minimum at around 19.5% yield and a subsequent increase at higher yield: experimental m/z, 111.039, 121.068, 125.061, 131.072, 135.091, 137.108, 149.107. A subset of Group B showed an initial decrease in PTR-MS signal intensity until yield 19.5% and then no changes at higher yield: 80.045, 97.025, 99.037, 107.047, 109.069, 117.044.

Group C (example Fig. 6c): compounds that show no clear trend: experimental m/z 55.054, 57.033, 57.069, 63.027, 71.046, 83.046, 85.058, 103.067, 110.055, 123.087.

Group D (example Fig. 6d): compound that shown an increase in headspace concentration with increasing yield: experimental m/z 127.034.

Unexpected results of the headspace PTR-MS analysis show that espresso extraction is not only simple diffusion of the compounds from the coffee particles into the solution, but other phenomena take place that need further and more detailed studies. Efficiency of espresso extraction decreases with increasing time^20^, and studies have suggested using faster extraction times for efficient extractions^18^. Only extraction (diffusion of VOCs) does not explain the measured headspace concentration of VOCs. We must consider other hypothetical phenomena that might happen before, during and after extraction: (i) Before extraction, starting from the grinding process aroma loses caused by the desorption of the VOCs from coffee matrix can happen. Presently there is little information about quantities of these losses in a short time scale of typical espresso preparation (< 1 min). (ii) During the extraction process, degassing of carbon dioxide from coffee causes formation of crema. Release of gas also transports some volatile compounds away into the surrounding atmosphere, preventing them to dissolve in the beverage^28^. (iii) Post extraction aroma losses occur from the flow of the espresso and by evaporation from the cup. Data by Sanchez et al.^6^ show that increasing extraction temperature increases the concentration of volatiles when measured by PTR-MS over the flow of espresso. This effect might not be because of better extraction at higher temperature, but due to higher evaporative losses from a flow of brew at higher temperatures. (iv) Matrix effects of the coffee brew at varying extraction yield can have an impact on the VOC partitioning between the brew and headspace producing the observed phenomena.

In conclusion, by varying the extraction time the aroma profile of an espresso is modulated in a non-linear way. “Turbo” espressi with faster than usual extraction time have the potential to produce more fruity-tasting beverages by better retaining highly volatile, non-polar aroma molecules.

### Sensory analysis

**Figure 7:**
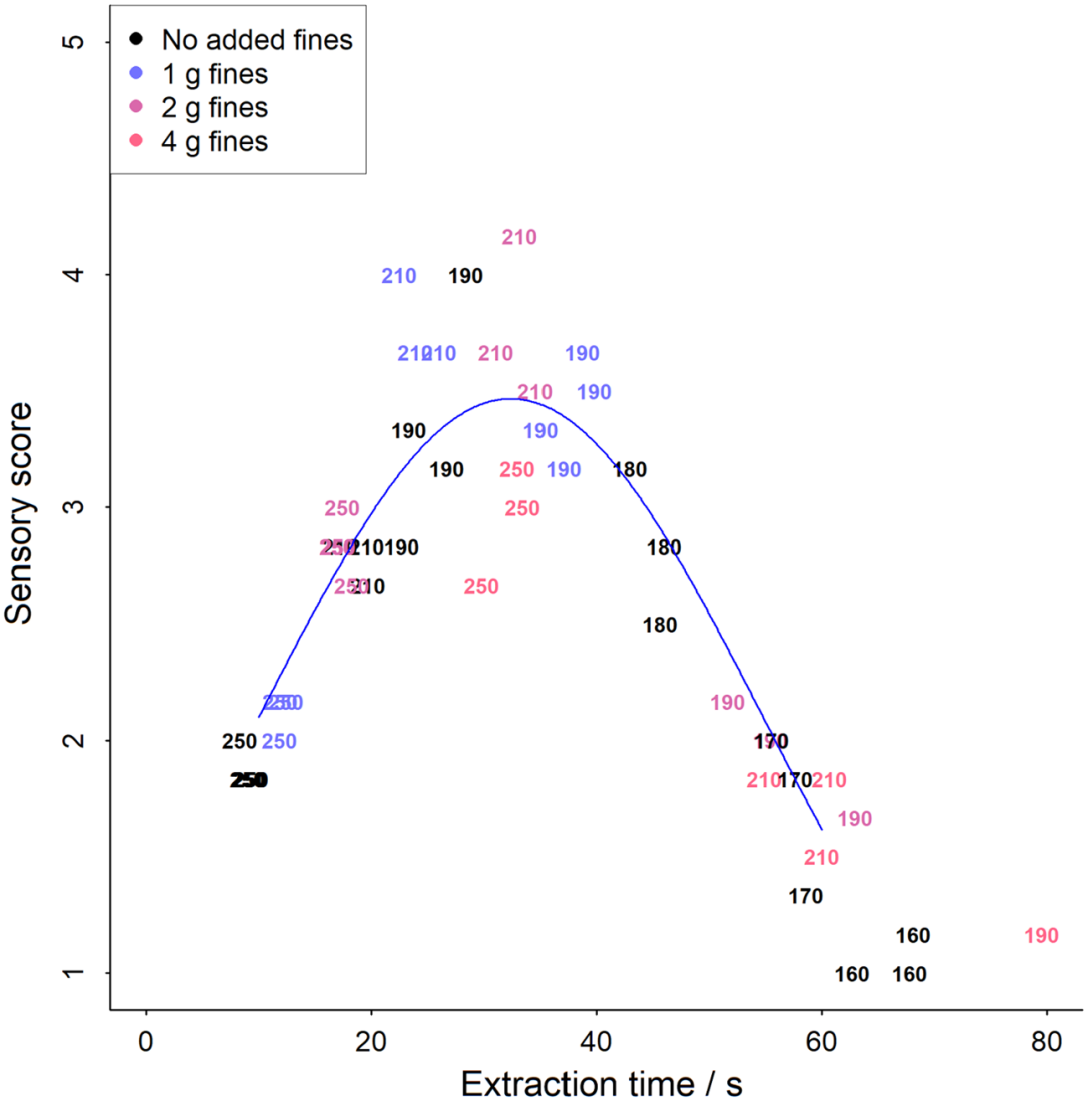
Total sensory analysis score of the extracted espresso coffees as a function of extraction time, extracted by varying the grind size and adding fines to the R&G coffee. The numbers on the chart denote the grinder setting, and the colour the amout of fines that was added, respectively.

Complimentary to the instrumental measurements, a hedonic approach to the sensory analysis has been tested to evaluate if an increase of fines in the cup leads to an undesirable flavor profile of an espresso. An experienced coffee taster has evaluated the coffee samples according to 3 basic espresso attributes (flavor, balance and tactile) and an average score of those has been generated. Such a process was used, because it is like the procedure to optimize extraction (colloquially known as “dial-in espresso”) in a specialty coffee shop. Figure 7 shows the scores as a function of extraction time and extraction yield. We did not observe any penalty in the sensory scores of the espresso when using R&G coffee with a higher share of fines. Surprisingly, two samples with 1 and 2 g of added fines, at the grinder setting of 210, were among the highest scoring espresso.

From the point of view of the panelist, optimal flavor profile for this coffee was when extracted around 30 s, and to 19–20% extraction yield. This is not in the range of previously discussed “turbo” espresso, hence we must consider that green coffee and roast profile used are the parameters that will impact at which extraction conditions a coffee will have its peak sensory quality. Since new roast styles and green coffee post-harvest processes have been emerging recently, information to draw clear general conclusions is presently not yet available. However, when considering optimization of efficient extraction by shortening extraction time, the methodology used this study can be used as a tool for a decision about how to optimize the extraction for high yield, short extraction time but keeping high flavor quality.

## Conclusions and outlook

The findings of this study confirm that the particle size distribution plays a principal role in espresso extraction and that a single value for particle size doesn’t fully describe expected extraction dynamics. The results show that, for a given coffee, the extraction time of an espresso can be predicted by knowing the share of fines and the size of the main particles. The effect of increased surface area due to smaller particle size for R&G coffee with high fines fraction on the extraction efficiency seems to be marginal. This leads to the conclusion that the fines are from a practical standpoint only modifying coffee bed permeability, when extracting within the range of variables tested in this study. The next step that needs studying regarding R&G PSD is the shape and the width of the main peak in the PSD. There is however to our knowledge presently no laboratory scale coffee grinder available that could vary systematically the width of the main PSD peak.

We provide evidence that losses of volatile compounds occur before or during extraction (from R&G coffee and from the beverage as it is being extracted) and extraction efficiency impacts the final aroma composition in the cup. Fast low yield extractions contain higher contents of highly volatile and non-polar VOCs, compared to slower extractions with higher yield, possible due to higher post-extraction losses from longer extraction times. The relation of coffee aroma to extraction yield is non-linear and fast extraction times may be preferred.

### Supplementary Information

Supplementary Figure S1.

## Data Availability

The datasets used and/or analysed during the current study available from the corresponding author on reasonable request.
