# Supplementary material for: The role of fines in espresso extraction dynamics
Source: Sci Rep. 2024 Mar 7;14:5612. doi: 10.1038/s41598-024-55831-x (PMC10920694; doi:10.1038/s41598-024-55831-x)

Supplementary Figure S1: Flow rates of espressi extracted by varying the grind size and share of fines (grinder setting 250 solid lines, 210 dashed lines, 190 dotted lines) and the amount of added fines (no added fines yellow, 1 g magenta, 2 g purple, 4 g blue). The flow rates were calculated by the derivative of the dynamic beverage weights. Each extraction of the replicates (n=3) is displayed. The addition of fines does not appear to drastically or funamentally change the typical continously increasing flow rate profile of espresso extraction.
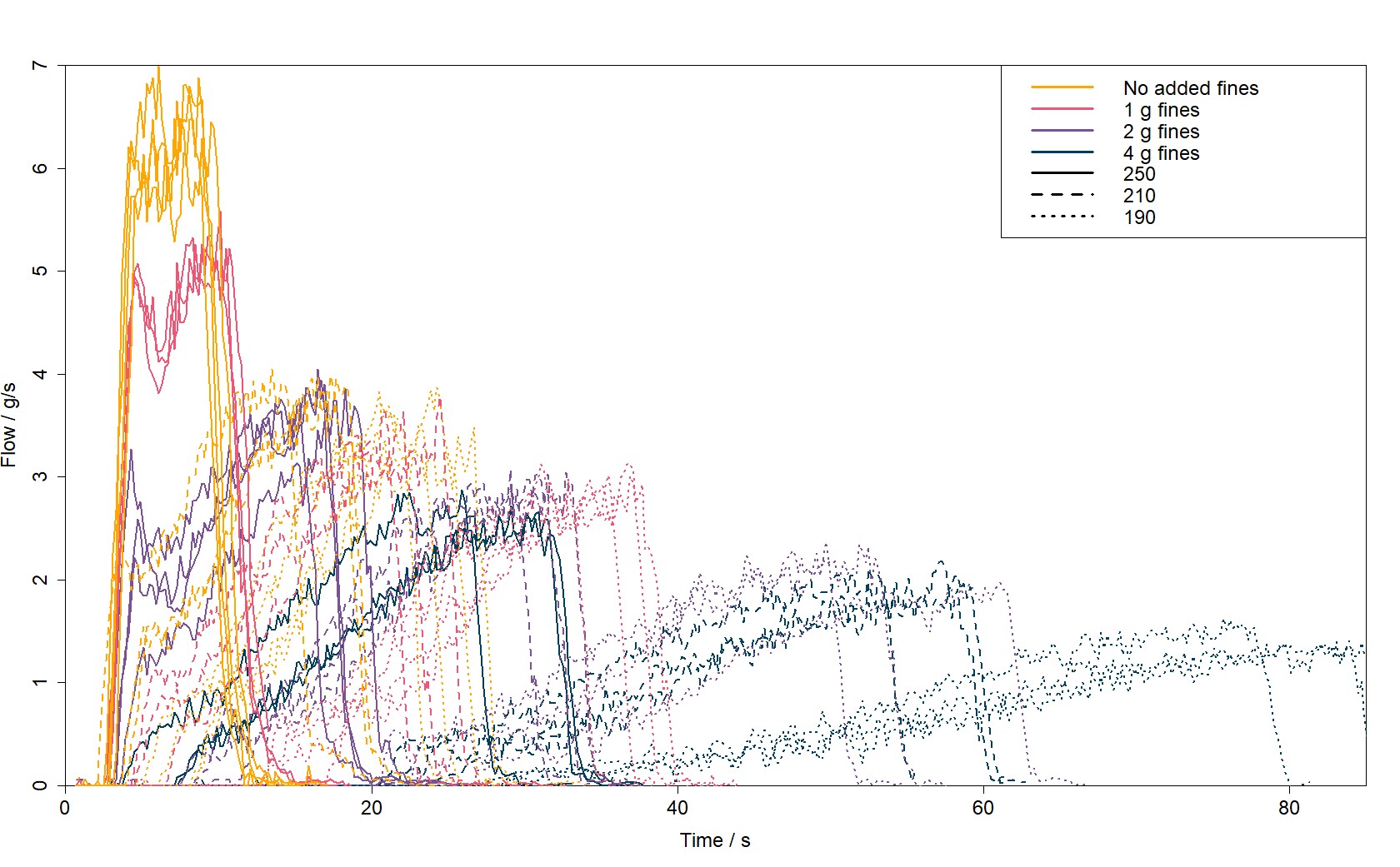

Supplement: Supplementary file 1 — Supplementary Figure S1. [file 41598_2024_55831_MOESM1_ESM.docx]
